# Structure–Activity Relationships (SARs) of α-Ketothioamides as Inhibitors of Phosphoglycerate Dehydrogenase (PHGDH)

**DOI:** 10.3390/ph13020020

**Published:** 2020-01-22

**Authors:** Quentin Spillier, Séverine Ravez, Judith Unterlass, Cyril Corbet, Charline Degavre, Olivier Feron, Raphaël Frédérick

**Affiliations:** 1 Medicinal Chemistry Research Group (CMFA), Louvain Drug Research Institute (LDRI), Université Catholique de Louvain, 1200 Brussels, Belgium; Quentin.Spillier@nyulangone.org (Q.S.); charline.degavre@student.uclouvain.be (C.D.); 2 Pole of Pharmacology and Therapeutics (FATH), Institut de Recherche Expérimentale et Clinique (IREC), Université Catholique de Louvain, 1200 Brussels, Belgium; cyril.corbet@uclouvain.be (C.C.); olivier.feron@uclouvain.be (O.F.); 3 UMR-S1172—JPArc—Centre de Recherche Jean-Pierre AUBERT Neurosciences et Cancer, Université de Lille, Inserm, CHU Lille, 59000 Lille, France; severine.ravez@univ-lille.fr; 4 Science for Life Laboratory, Department of Oncology-Pathology, Karolinska Institutet, 171 21 Solna, Sweden; judith_unterlass@web.de

**Keywords:** PHGDH, α-ketothioamides, serine synthesis pathway inhibitors

## Abstract

For many years now, targeting deregulation within cancer cells’ metabolism has appeared as a promising strategy for the development of more specific and efficient cancer treatments. Recently, numerous reports highlighted the crucial role of the serine synthetic pathway, and particularly of the phosphoglycerate dehydrogenase (PHGDH), the first enzyme of the pathway, to sustain cancer progression. Yet, because of very weak potencies usually in cell-based settings, the inhibitors reported so far failed to lay ground on the potential of this approach. In this paper, we report a structure–activity relationship study of a series of α-ketothioamides that we have recently identified. Interestingly, this study led to a deeper understanding of the structure–activity relationship (SAR) in this series and to the identification of new PHGDH inhibitors. The activity of the more potent compounds was confirmed by cellular thermal shift assays and in cell-based experiments. We hope that this research will eventually provide a new entry point, based on this promising chemical scaffold, for the development of therapeutic agents targeting PHGDH.

## 1. Introduction

The ability of cancer cells to reprogram their metabolism was demonstrated in numerous studies over the past two decades [1,2,3]. These metabolic modifications are used by cancer cells in order to proliferate in harmful environments (hypoxic zones, deficiencies in the external supply of glucose and amino acids) by producing certain metabolites and antioxidants more efficiently. It quickly became apparent that the development of treatments specifically targeting these deregulated metabolic pathways would reduce tumor development while significantly limiting the side effects associated with conventional non-targeted therapies [4].

In this purpose, among the many pathways studied, the serine synthesis pathway appeared to be a promising target [5,6]. Indeed, this pathway plays a central role in one-carbon metabolism, methylation processes, and allows the cells to produce the building blocks required for their proliferation [7]. Serine can also be used to produce glutathione and therefore offers resistance against the oxidative stress associated with intense proliferation [8]. In addition, the three enzymes constituting this pathway (phosphoglycerate dehydrogenase (PHGDH), PSAT-1, and PSPH) are overexpressed in many cancer types (breast cancer, melanoma, glioma) and often associated with poor prognosis in the patient [9].

Phosphoglycerate dehydrogenase (PHGDH) is the first enzyme to catalyze the rate-limiting step of the serine synthesis pathway. Recently, its study highlighted key roles in diverting glycolytic flow to the serine pathway leading to a tumor invasion process. PHGDH is overexpressed and/or genomically amplified in more than 16% of cancer lines [10]. Moreover, the overexpression of this protein appears to lead to greater metastatic invasion in animal models and could also be correlated with greater aggressiveness and lower survival rates [11,12]. Finally, it was demonstrated that inhibition or silencing of this enzyme can selectively impact tumor growth while sparing healthy cells [13]. Therefore, in recent years, there has been an increased interest in the development of small molecule PHGDH inhibitors.

**Figure 1:**
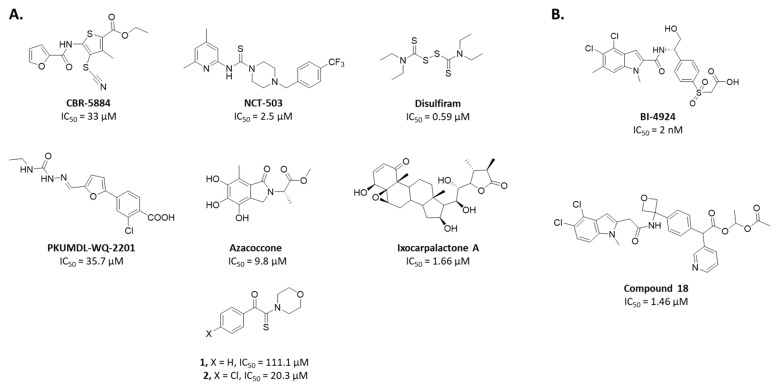
Representative phosphoglycerate dehydrogenase (PHGDH) inhibitors and their IC_50_. (**A**) Allosteric inhibitors. (**B**) NAD competitive inhibitors.

Since 2015, several chemical series were identified to inhibit PHGDH enzymatic activity (Figure 1). Two of them are NAD^+^-competitive and thus interact with the Rossmann fold (BI-4924 and compound **18**). These compounds exhibit PHGDH inhibition in the submicromolar range in isolated enzyme assays but have poor cellular efficacy notably because of the competition with a high intracellular NAD^+^ concentration [14,15]. The other series of inhibitors (CBR-5884, NCT-503, disulfiram, Azacoccone, PKUMLD-WQ-220, Ixocarpolactone) have an allosteric mode of action, and although less effective than the NAD^+^-competitive compounds, they are usually characterized by a better inhibition profile on cancer cell lines in in vitro and in vivo xenograft models. However, for most of these allosteric inhibitors, their precise sites of action are yet to be determined or have been concluded from docking studies so far [16,17,18,19,20,21].

Despite the interest in targeting PHGDH and the increasing number of reported inhibitors, the weak in cellulo and in vivo efficacy of the inhibitors hamper the understanding of the role PHGDH plays in cancer progression. To date, the developed compounds were mainly used to study the serine synthesis pathway in restoring the sensitivity of cancer cells to conventional chemotherapy (bortezomib, doxorubicin, sorafenib) [22,23,24]. The development of more effective PHGDH inhibitors to investigate the serine synthesis pathway in cancer biology hence remains necessary.

In 2017, our group identified a series of PHGDH inhibitors based on an α-ketothioamide motif and endowed with micromolar inhibitory potency (Figure 1A, compounds **1** and **2**). [25] Although these compounds remain only moderately active on the isolated target, they are characterized by an excellent ligand efficiency (LE) of around 0.34. Moreover, these molecules demonstrated their ability to reduce the proliferation of cancer cell lines overexpressing PHGDH (SiHa, HL-60) without affecting proliferation in cell lines with little or no expression of this enzyme (MDA-MB-231, HL-60 shPHGDH). The study also identified these molecules as irreversible and non-competitive PHGDH inhibitors [25]. Preliminary structure–activity relationships (SARs) were undertaken and revealed that substituents, notably halogens, introduced in the *para* position enhanced PHGDH inhibition whereas *ortho-* or *meta*- substitutions led to a decrease of the PHGDH inhibitory potency. Since α-ketothioamides proved to be useful as PHGDH inhibitors, we put significant effort to explore the SARs around this series.

## 2. Results and Discussion

Compound **1** is characterized by an aryl group (part A) and a morpholine moiety (part C) that are connected by an α-ketothioamide linker (part B) (Figure 1). In this manuscript, the SAR study was investigated by: (1) modulation of the aromatic moiety or by insertion of alkyl groups on part A; (2) introduction of different amino moieties on part C; and (3) variation of the nature of the linker (part B). As a result, a library of 39 compounds was designed, synthesized, and evaluated in vitro using a PHGDH coupled assay at a single compound concentration of 150 µM.

### 2.1. Chemistry

**Scheme 1:**
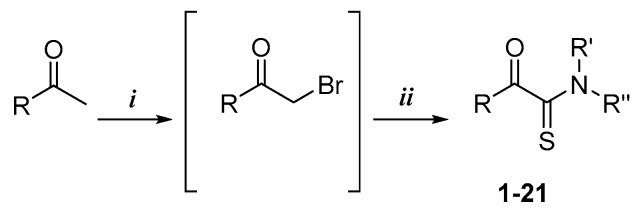
Synthetic pathway of α-ketothioamide derivatives **1**–**21**, **38**, and **39** ^a^. ^a^ Reagents and conditions: (**i**) Br_2_, CHCl_3_, TBAB, r.t., for 2 h; (**ii**) S_8_, appropriated amino group, DMF, r.t., 8%–73%. R represents aryl or alkyl moieties and R’ and R’’ represent different amino group as detailed in § 2.3 SAR’s Investigation.

The α-ketothioamides **1**–**21** were obtained using a two-step procedure involving the bromination of the corresponding acetone (1 equiv.) derivatives when necessary in the presence of bromine (1.2 equiv.) and tetra-n-butylammonium bromide (TBAB) in chloroform (Scheme 1). The resulting product or the commercially available brominated compound was engaged in the Willgerodt–Kindler reaction without any further purification according to a reported procedure. [25] DMF, octasulfur (1.5 equiv.), and appropriated amine (3 equiv.) were introduced into the reaction mixture and the reaction proceeded to completion at room temperature.

**Scheme 2:**
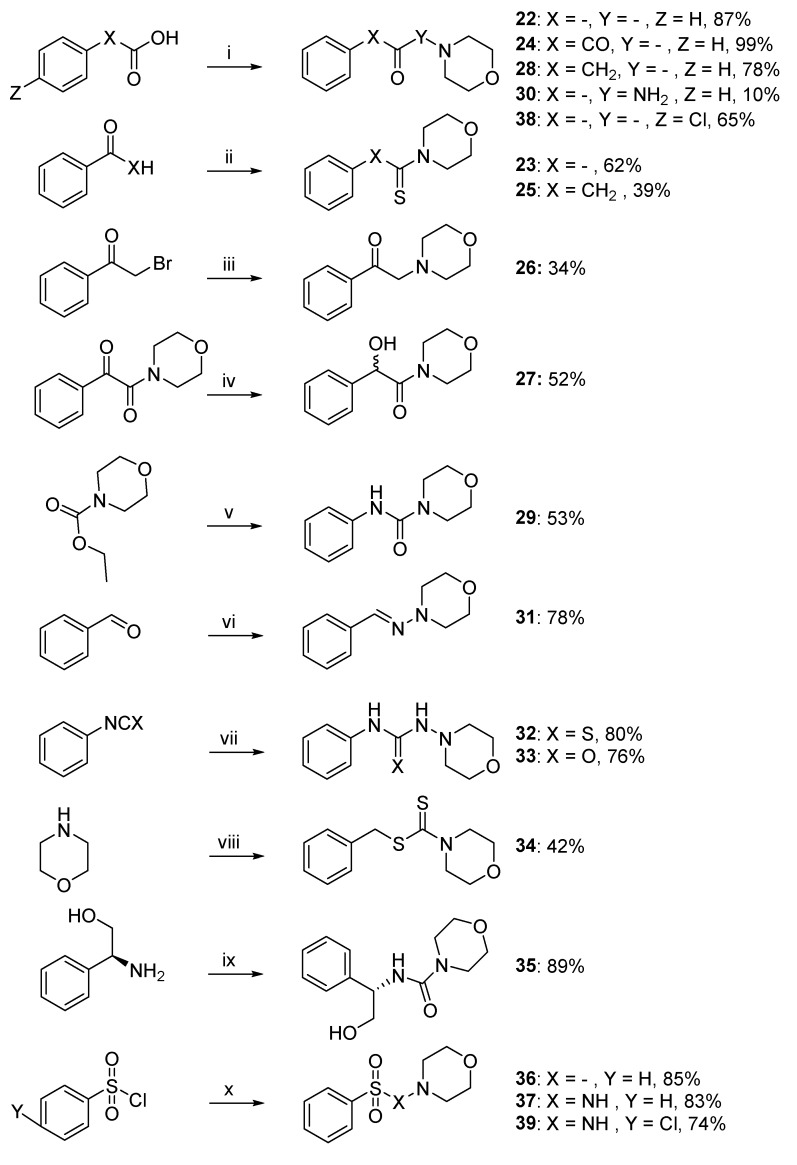
Synthetic pathway of compounds **22**–**39** ^a^. ^a^ Reagents and conditions: (**i**) COCl_2_, DIPEA, appropriated amine, CH_2_Cl_2_, r.t., 10%–99%; (**ii**) S_8_, morpholine, DMF, 39%–62%; (**iii**) morpholine, K_2_CO_3_, MeCN, r.t., 34%; (**iv**) NaBH_4_, MeOH, r.t., 52%; (**v**) (1) POCl_3_, MeCN, 50 °C, (2) aniline, DIPEA, 0 °C to r.t., 53%; (**vi**) 4-amino-morpholine, MgSO_4_, CH_2_Cl_2_, r.t., 78%; (**vii**) 4-amino-morpholine, CH_2_Cl_2_, r.t., 76%–80%; (**viii**) (1) CS_2_, Et_2_O, r.t., (2) (thiocyanatomethyl)benzene, ACN, r.t., 42%; (**ix**) morpholine-4-carbonyl chloride, DIPEA, CH_2_Cl_2_, r.t., 89%. (x) Appropriated amine, Et_3_N, r.t., 83%–85%.

For the modification of the central linker, syntheses of compounds are outlined in Scheme 2. Compounds **22**, **24**, **28**, and **30** were synthesized by nucleophilic acyl substitution. In the first step, different carboxylic acids were acylated with oxalyl chloride to obtain the corresponding acid chlorides. These chlorides then reacted with a morpholine molecule, or 4-amino-morpholine in the case of compound **30**, to obtain the desired compounds. Benzaldehyde or acetophenone were used in the Willgerodt–Kindler reaction described above to lead to the synthesis of compounds **23** and **25**. The compound **26** was obtained thanks to an SN2-type reaction between morpholine and bromo-acetophenone and the alcohol **27** was obtained after the NaBH_4_ reduction of **24**. The reaction of commercially available ethyl morpholine-4-carboxylate with POCl_3_ afforded the corresponding acyl chloride, which was converted to compound **29** by reaction with aniline. The reaction of 4-aminomorpholine and benzaldehyde furnished the desired methylethanimine derivative **31**. The reaction of morpholine with isothiocyanatobenzene or isocyanatobenzene afforded the hydrazinecarbothioamide **32** and the urea derivative **33**, respectively, with good yield (>76%). Compound **34** was synthesized by a two-step reaction. Firstly, CS_2_ and morpholine were used in order to produce the corresponding morpholinium salt. Then, this salt was reacted with (thiocyanatomethyl)benzene in acetonitrile to afford the desired derivative **34**. Coupling between the commercially available alcohol and morpholine-4-carbonyl chloride furnished the desired compound **35**. Finally, the reaction of morpholine or 4-amino-morpholine with a benzenesulfonyl chloride generated compounds **36** and **37**.

### 2.2. PHGDH Biochemical Assay Optimization

**Figure 2:**
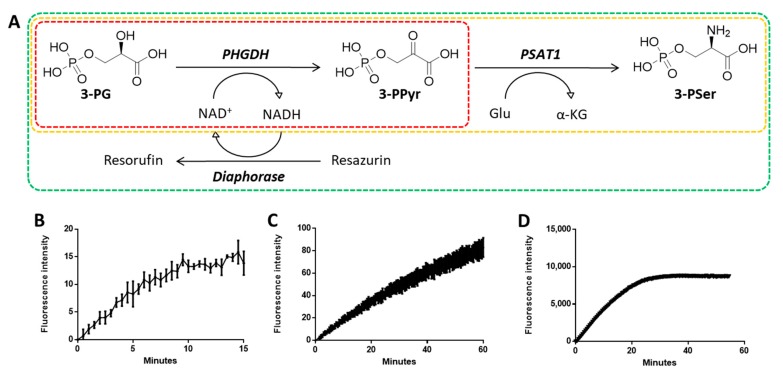
PHGDH coupled biochemical assay system. (**A**) Schematic representation of the coupled system. Continuous measurement of PHGDH activity for initial PHGDH assay (**B**), for PHGDH/PSAT1 coupled assay (**C**), and for present fully coupled assay (**D**). (3-PG, 3-phosphoglycerate; 3-PPyr, 3-phosphopyruvate; 3-PSer, 3-phosphoserine).

Prior to inhibitor evaluation, we undertook the optimization of our biochemical assay in order to achieve reliable results. Our initial PHGDH assay contained solely the dehydrogenase and its substrates; the enzyme activity was quantified by directly measuring NADH fluorescence, hence yielded a low fluorescent signal (Figure 2B).

PHGDH activity was monitored by measuring a continuous increase of fluorescence (Ex 544 nm/Em 590 nm) in a coupled-enzyme system including three enzymes: PHGDH, PSAT1, and diaphorase (Figure 2A). The addition of PSAT1 promotes accumulation of NADH by the PHGDH reaction and generates a significant increase in the fluorescent signal (Figure 2C). Moreover, by coupling PHGDH to diaphorase, which utilizes NADH to produce the fluorescent molecule resorufin from resazurin, the assay red-shifted into a spectral region that reduced interference from compounds, as well as greatly increased.

In optimized conditions, Michaelis–Menten constants were estimated for substrates of PHGDH (3-PG and NAD^+^) and substrates of PSAT1 (3-PSer and α-KG). K_m_ values for 3-PG, NAD^+^, 3-PSer, and α-KG were determined to be 294.3, 30.4, 25.1, and 35 µM, respectively (Table 1).

The optimized fully coupled assay provides robust measurement of PHGDH activity, suggesting it was amenable to evaluation of our inhibitors.

### 2.3. SARs Investigation

Although a structural variety has been introduced at part A of the α-ketothioamide scaffold in our previous work, the substitution by alkyl groups has not been previously studied in detail. Hence, we began our PHGDH SAR investigations with a focus on part A. As depicted in Table 2, only aryl groups led to PHGDH inhibition (**1**, **2**, and **4**) whereas the introduction of alkyl groups in this position resulted in inactive derivatives. Moreover, substitutions on the aryl moiety are only tolerated in the *para* position with the chlorinated derivative **2** being the most potent.

To expand the SARs, the introduction of different amino moieties on part C was undertaken keeping fixed parts A and B (Table 3). Except for the 4-methylpiperidine analog (**14**), the introduction of a bulky group in part C of the molecule abolished the inhibitory potency.

Subsequently, we shifted our SAR analysis to the linker portion B (Table 4). This feature of the pharmacophore proved essential to activity as the replacement led to significant loss of PHGDH activity (IC_50_ > 150 µM). However, two modifications to this region of the molecule tolerated such shortening of the linkage from the thiocarbonyl (**23**, IC_50_ = 106 µM) or modification by sulfonohydrazide (**37**, IC_50_ = 92 µM).

**Figure 3:**
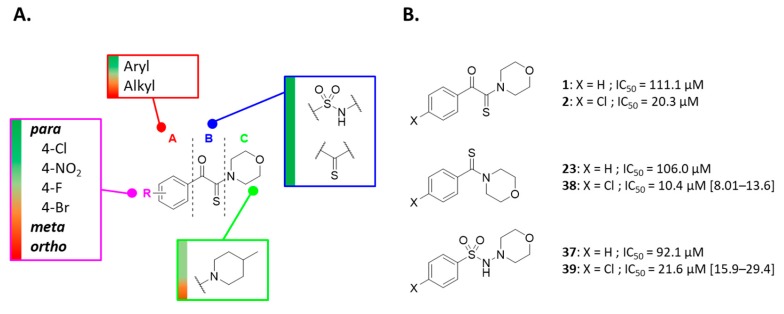
Overview of the structure−activity relationships. Color code: green > yellow > red, for decreasing PHGDH activity (**A**). Influence of the *para*-chloro substitution on the potency of PHGDH inhibition (**B**). Under brackets: standard deviation in µM.

Based on the modification of the linker portion, two 4-chloroderivatives carrying a thiocarbonyl (**38**) or a sulfonohydrazide (**39**) instead of a ketothioamide linker were designed and synthesized (Scheme 2). As expected, substitution of the *para* position by a chlorine atom resulted in an improvement in PHGDH inhibition (Figure 3B). The most promising compounds (**1**, **2**, **23**, **38**, **37**, and **39**) were validate in a counter screen against PSAT1 and the diaphorase. Based on the preliminary study reported earlier and the present work, the following SARs can be highlighted (Figure 3A).

### 2.4. PHGDH Engagement Investigated by Cellular Thermal Shift Assay (CETSA)

**Figure 4:**
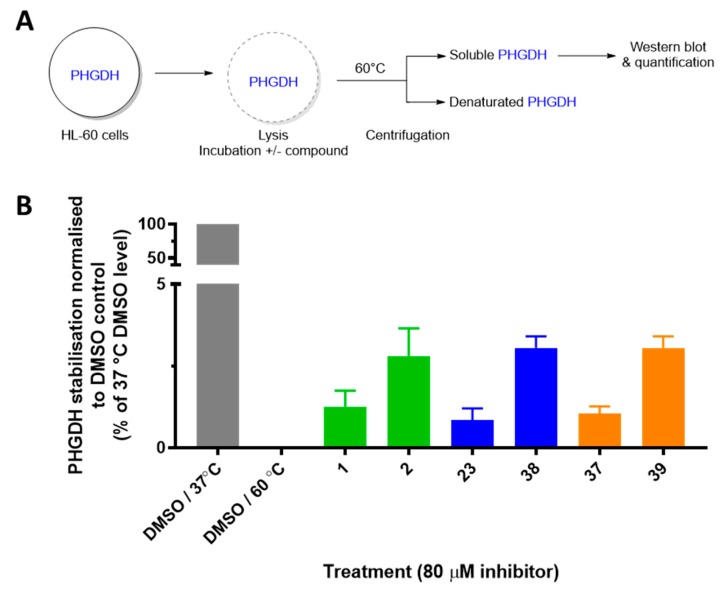
Cellular thermal shift assay (CETSA) for the most promising inhibitors in HL-60 cells. (**A**) Schematic overview of CETSA experiment. (**B**) Quantification of thermostable PHGDH normalized to the respective control at 37 °C. Results represent the average of two independent experiments.

Next, in order to confirm that the most potent inhibitors target PHGDH in more complex biological models, we measured their ability to stabilize PHGDH in cell lysates using the cellular thermal shift assay (CETSA). In this assay, HL-60 (leukemia cancer cells) lysates were incubated with DMSO or with 80 µM of the inhibitor. The lysate was then heated to a pre-determined temperature, at which most of PHGDH was denaturated (60 °C). The fraction of non-denatured PHGDH was quantified by Western blot and allowed to identify the compounds that bind and stabilize PHGDH. The percentages of stabilization compared to the DMSO control and non-denatured PHGDH (heated to 37 °C) of the six most potent PHGDH inhibitors are depicted in Figure 4.

While in untreated cells PHGDH was denaturated at 60 °C, the addition of compounds **1**, **23**, and **37** leads to a small stabilization of the protein (1.2%, 0.8%, and 1.1%, respectively) thus proving the interaction between these compounds and PHGDH in cell lysates. More interestingly, the *para*-chlorinated derivatives (**2**, **38**, and **39**) present a greater stabilization of the protein compared to the non-halogenated compounds (2.8%, 3.1%, and 3.2% respectively); hence, this is in agreement with increased PHGDH inhibition in the enzyme activity assay.

### 2.5. Cell-Based Evaluation

We assessed the ability of *para*-chlorinated derivatives, **38** and **39**, to inhibit tumor proliferation. To this end, breast cancer cells overexpressing PHGDH (BT-20), breast cancer cells that do not express PHGDH (MDA-MB-231) and healthy fibroblast cells as control (BJ-5ta) were treated with compounds **1**, **2**, **38**, **39** and the reference inhibitor NCT-503, used for comparison purpose. The IC_50_ values determined are depicted on Table 5.

Except for compound **39**, which was deprived of any inhibition of tumor proliferation up to 100 µM probably due to poor membrane permeability, compounds **1**, **2**, and **38** selectively inhibit BT-20 growth without affecting healthy BJ-5ta or the PHGDH-independent tumor cell line, MDA-MB-231. Interestingly, these inhibitions were similar to the one observed with NCT-503. Again, these results demonstrate the potential of this compound class as a starting point for the design of new therapeutic agents targeting PHGDH.

## 3. Conclusions

The development of new PHGDH inhibitors is emerging as a promising strategy to develop new targeted therapy. We previously reported the discovery of compound **1**, a PHGDH inhibitor with robust cancer cell inhibition. Here, we have detailed the SARs investigation around this α-ketothioamide motif. Replacement of the aryl and modulation of the amino groups were not tolerated in terms of PHGDH inhibition. However, modification of the ketothioamide linker by a thiocarbonyl or by a sulfonohydrazide led to the identification of novel inhibitors. As expected, the addition of a chlorine atom in *para* position on the most promising molecules resulted in the discovery of novel micromolar range inhibitors (**38** and **39**). Target engagement was confirmed in cellular thermal shift assay (CETSA) and the anticancer potential was validated in cell-based experiments. Interestingly, the identification of the sulfonohydrazide motif as a new series of inhibitors of this enzyme offers interesting prospects in the development of future PHGDH inhibitors, this function being reported in several works as non-toxic [29,30]. It should be noted that recent mass spectrometry studies suggest that some of the inhibitors discussed here would interact at the c-terminal end of PHGDH. This will be detailed in a forthcoming publication. 

## 4. Materials and Methods

### 4.1. General Chemistry

All reagents were purchased from chemical suppliers and used without purification. Thin-layer chromatography (TLC) was performed using silica gel 60 F254 plates, with observation under UV when necessary. Melting points were recorded on an Electrothermal IA9000 melting point system. ^1^H NMR spectra were recorded on an AVANCE II 400 MHz Bruker spectrometer with CDCl_3_ or DMSO-d_6_ as the solvent. ^13^C NMR spectra were recorded at 100 MHz. All coupling constants were measured in hertz (Hz), and the chemical shifts (δH and δC) were quoted in parts per million (ppm) relative to TMS (δ0), which was used as the internal standard. Data were reported as follows: chemical shift, multiplicity (s = singlet, d = doublet, t = triplet, q = quartet, br = broad, m = multiplet), integration, and coupling constant (Hz). High-resolution mass spectroscopy (HRMS) analyses were carried out on an LTQ-Orbitrap XL hybrid mass spectrometer (Thermo Fisher Scientific, Bremen, Germany). Data were acquired in positive ion mode using full-scan MS with a mass range of 100–1000 *m*/*z*. The orbitrap resolution was set at 30,000 (fwhm definition). All experimental data were acquired using daily external calibration prior to data acquisition. Appropriate tuning of the electrospray ion source was done. The following electrospray inlet conditions were applied: flow rate, 100 μL/min; spray voltage, 5 kV; sheath gas (N_2_) flow rate, 20 arbitrary unit (au); auxiliary gas (N_2_) flow rate, 10 au; capillary temperature, 275 °C; capillary voltage, 45 V; tube lens, 80 V. High-performance liquid chromatography (HPLC) analyses were performed on an LC system using a YMC-Triart C-18 (250 mm × 4.6 mm, 5 μm) column as the stationary phase. Mobile phase contained water/CH_3_CN (30:70, *v*/*v*) and was maintained isocratically at the flow rate of 1 mL/min. The column temperature was maintained at room temperature. The peaks were monitored at a wavelength of 215 nm. The purity of all compounds tested was greater than 95%, as determined by HPLC and ^1^H NMR.

Compounds **1**–**11** were synthesized according to General Procedure I.

#### 4.1.1. General Procedure I

To a stirred solution of ethanone derivative (1 equiv.) in chloroform was added dropwise a solution of dibromine (1.2 equiv.) in chloroform. After 2 h, the solvent was evaporated in vacuo to give a crude oil consisting mainly of 2-bromo-1-(substituted)-ethanone compound along with trace amounts of 2,2-dibromo-1-(substituted)-ethanone compound. The mixture was used without purification in the next step. To the synthesized or commercial 2-bromo-1-(substituted)-ethanone derivative were added, in sequence, DMF, cyclooctasulfur (1.5 equiv.), and morpholine (3 equiv.). The mixture was then stirred at room temperature. After completion, the reaction mixture was quenched with distilled water to give a precipitate, which was further washed with distilled water. The residue was recrystallized or purified by silica gel chromatography if necessary.

2-Morpholino-1-phenyl-2-thioxoethan-1-one (**1**). Acetophenone (2.00 g, 16.60 mmol) and dibromine (1.01 mL, 19.90 mmol) were mixed in chloroform (15 mL) to obtain the 2-bromo-1-phenyl-ethanone, and this intermediate was reacted in a second time with morpholine (4.34 mL, 49.80 mmol) and sulfur (0.79 g, 24.90 mmol) in DMF (10 mL). Methanol was used for recrystallization to afford the title compound (2.85 g, 73%). R*_f_* 0.2 (cyclohexane/EtOAc: 8/2). M.p.: 110–112 °C. ^1^H NMR (400 MHz, CDCl_3_): *δ*H (ppm) 3.59–3.62 (t, 2H, *J* = 4.8 Hz), 3.69–3.71 (t, 2H, *J* = 4.8 Hz), 3.90–3.92 (t, 2H, *J* = 4.8 Hz), 4.33–4.35 (t, 2H, *J* = 4.8 Hz), 7.48–7.52 (m, 2 ArH), 7.59–7.65 (m, 1 ArH), 7.99–8.01 (d, 2 ArH, *J* = 8.2 Hz). ^13^C NMR (100 MHz, CDCl_3_): *δ*C (ppm) 47.13, 51.95, 66.40, 66.52, 128.99 (2C), 129.86 (2C), 133.26, 134.48, 187.90 (C=O), 195.70 (C=S). HRMS (ESI^+^): *m/z* calcd for C_12_H_13_NO_2_S [M + H]^+^ 236.0739, found 236.0737.

1-(4-Chlorophenyl)-2-morpholino-2-thioxoethan-1-one (**2**). This compound was synthesized according to General Procedure I using 2-bromo-1-(2-chlorophenyl)ethanone (0.50 g, 2.14 mmol), morpholine (0.56 mL, 6.42 mmol), and sulfur (0.10 g, 3.21 mmol) in DMF (10 mL). Acetonitrile was used for recrystallization to afford the title compound as a yellow solid (0.22 g, 38%). R*_f_* 0.2 (cyclohexane/EtOAc: 8/2). M.p.: 135–137 °C. ^1^H NMR (400 MHz, CDCl_3_): *δ*H (ppm) 3.58–3.71 (m, 4H), 3.89–3.92 (t, 2H, *J* = 4.8 Hz), 4.31–4.33 (t, 2H, *J* = 4.8 Hz), 7.46–7.48 (d, 2 ArH, *J* = 8.8 Hz), 7.93–7.95 (d, 2 ArH, *J* = 8.6 Hz). ^13^C NMR (100 MHz, CDCl_3_): *δ*C (ppm) 47.18, 51.97, 66.39, 66.53, 129.36 (2C), 131.22 (2C), 131.74, 141.06, 186.45 (C=O), 194.94 (C=S). HRMS (ESI^+^): *m/z* calcd for C_12_H_13_ClNO_2_S [M + H]^+^ 270.0350, found 270.0350.

1-(3-Chlorophenyl)-2-morpholino-2-thioxoethan-1-one (**3**). This compound was synthesized according to General Procedure I. 1-(3-Chlorophenyl)ethanone (2.00 g, 12.90 mmol) and dibromine (0.78 mL, 15.50 mmol) were mixed in chloroform (15 mL) to obtain the 2-bromo-1-(3-chlorophenyl)-ethanone and this intermediate was reacted in a second time with morpholine (3.38 mL, 38.80 mmol) and sulfur (0.62 g, 19.40 mmol) in DMF (10 mL). The residue was purified by silica gel chromatography (cyclohexane/EtOAc: 8/2) and the obtained oil was collected by filtration with diethyl ether to give the title compound as a yellow solid (1.50 g, 43%). R*_f_* 0.2 (cyclohexane/EtOAc: 8/2). M.p.: 92–94 °C. ^1^H NMR (400 MHz, CDCl_3_): *δ*H (ppm) 3.59–3.61 (t, 2H, *J* = 4.8 Hz), 3.70–3.72 (t, 2H, *J* = 4.8 Hz), 3.90–3.92 (t, 2H, *J* = 4.8 Hz), 4.31–4.33 (t, 2H,*J* = 4.8 Hz), 7.42–7.46 (dd, 1 ArH, *J* = 7.8 Hz), 7.57–7.59 (ddd, 1 ArH, *J* = 8.0 and 1.0 Hz), 7.85–7.87 (ddd, 1 ArH, *J* = 7.8 and 1.4 Hz), 7.96–7.97 (dd, 1 ArH, *J* = 1.8 Hz).^13^C NMR (100 MHz, CDCl_3_): *δ*C (ppm) 47.21, 52.00, 66.39, 66.52, 127.98, 129.61, 130.28, 134.33, 135.05, 135.30, 186.07 (C=O), 194.60 (C=S). HRMS (ESI^+^): *m/z* calcd for C_12_H_12_ClNO_2_S [M + H]^+^ 270.0277, found 270.0278.

1-(2,4-Dichlorophenyl)-2-morpholino-2-thioxoethan-1-one (**4**). This compound was synthesized according to General Procedure I using 2-bromo-1-(2,4-dichlorophenyl)-ethanone (1.00 g, 3.76 mmol), morpholine (0.99 mL, 11.28 mmol), and sulfur (0.18 g, 5.64 mmol) in DMF (10 mL). Acetonitrile was used for recrystallization to afford the title compound as a yellow solid (0.31 g, 28%). R*_f_* 0.4 (cyclohexane/EtOAc: 8/2). M.p.: 116–118 °C. ^1^H NMR (400 MHz, CDCl_3_): *δ*H (ppm) 3.75–3.77 (t, 2H, *J* = 4.8 Hz), 3.77–3.79 (t, 2H, *J* = 4.8 Hz), 3.89–3.91 (t, 2H, *J* = 4.8 Hz), 4.10–4.12 (t, 2H, *J* = 4.8 Hz), 7.30 (dd, 1 ArH, *J* = 2.6 and 8.4 Hz), 7.38 (d, 1 ArH, *J* = 2.6 Hz), 7.80 (d, 1 ArH, *J* = 8.4 Hz). ^13^C NMR (100 MHz, CDCl_3_): *δ*C (ppm) 47.68, 52.11, 65.95, 66.03, 127.83, 130.49 (2C), 133.08, 133.56, 139.55, 183.73 (C=O), 195.19 (C=S). HRMS (ESI^+^): *m/z* calcd for C_12_H_12_Cl_2_NO_2_S [M + H]^+^ 303.99603, found 303.99607.

1-Cyclohexyl-2-morpholino-2-thioxoethan-1-one (**5**). This compound was synthesized according to General Procedure I using 1-cyclohexylethanone (2.00 g, 15.86 mmol) and dibromine (0.96 mL, 19.03 mmol) mixed in chloroform (20 mL) to obtain the 2-bromo-1-cyclohexylethanone and this intermediate was reacted in a second time with morpholine (4.18 mL, 47.58 mmol) and sulfur (0.76 g, 23.79 mmol) in DMF (10 mL). The residue was purified by silica gel chromatography (Cyclohexane/EtOAc: 8/2) to give the title compound as a brown solid (0.58 g, 15%). R*_f_* 0.3 (Cyclohexane/EtOAc: 8/2). M.p.: 57–59 °C. ^1^H NMR (400 MHz, CDCl_3_): *δ*H (ppm) 1.18–1.37 (m, 5H), 1.69–1.99 (m, 5H), 3.22 (m, 1H), 3.61–3.62 (m, 2H), 3.73–3.74 (t, 2H, *J* = 4.8 Hz), 3.82–3.84 (t, 2H, *J* = 4.8 Hz), 4.19–4.21 (t, 2H, *J* = 4.8 Hz). ^13^C NMR (100 MHz, CDCl_3_): *δ*C (ppm) 25.52 (2C), 25.71, 28.10 (2C), 47.28, 47.49, 52.07, 66.31, 66.60, 197.85 (C=O, 201.05 (C=S). HRMS (ESI^+^): *m/z* calcd for C_12_H_20_NO_2_S [M + H]^+^ 242.1209, found 242.1208.

2-Morpholino-1-(pyridin-4-yl)-2-thioxoethan-1-one (**6**). This compound was synthesized according to General Procedure I using 2-bromo-1-(pyridine-4-yl)ethanone (0.50 g, 1.79 mmol), morpholine (0.47 mL, 5.37 mmol), and sulfur (0.08 g, 2.68 mmol) in DMF (10 mL). The residue was purified by silica gel chromatography (CH_2_Cl_2_/EtOAc: 5/5) to give the title compound as a yellow solid (0.15 g, 36%). R*_f_* 0.3 (CH_2_Cl_2_/EtOAc: 5/5). M.p.: 96–98 °C. ^1^H NMR (400 MHz, CDCl_3_): *δ*H (ppm) 3.60–3.63 (m, 2H), 3.71–3.74 (m, 2H), 3.91–3.93 (m, 2H), 4.31–4.34 (m, 2H), 7.77–7.79 (m, 2 ArH), 8.83–8.85 (m, 2 ArH). ^13^C NMR (100 MHz, CDCl_3_): *δ*C (ppm) 45.30, 50.05, 64.40, 64.56, 120.26 (2C), 137.86, 149.10 (2C), 183.37 (C=O), 191.58 (C=S). HRMS (ESI^+^): *m/z* calcd for C_11_H_13_N_2_O_2_S [M + H]^+^ 237.06922, found 237.06923.

1-(3,4-Dichlorophenyl)-2-morpholino-2-thioxoethan-1-one (**7**). This compound was synthesized according to General Procedure I using 2-bromo-1-(3,4-dichlorophenyl)ethan-1-one (1.00 g, 3.73 mmol), morpholine (0.96 mL, 11.2 mmol), and sulfur (0.18 g, 5.60 mmol) in DMF (15 mL). The residue was purified by silica gel chromatography (Cyclohexane/EtOAc: 3/2) to give the title compound as a yellow solid (0.32 g, 28%). R*_f_*0.6 (Cyclohexane/EtOAc: 3/2). M.p.: 134–136 °C. ^1^H NMR (400 MHz, CDCl_3_): *δ*H (ppm) 3.60–3.61 (m, 2H), 3.71–3.72 (m, 2H), 3.90–3.93 (m, 2H), 4.31–4.33 (m, 2H), 7.55–7.60 (m, 1 ArH), 7.79–7.84 (m, 1 ArH), 8.07–8.09 (m, 1 ArH). ^13^C NMR (100 MHz, CDCl_3_): *δ*C (ppm) 44.87, 49.63, 63.99, 64.15, 126.38, 128.69, 129.11, 130.76, 131.36, 136.75, 182.57 (C=O), 191.61 (C=S). HRMS (ESI^+^): *m/z* calcd for C_12_H_12_Cl_2_NO_2_S (M + H)^+^ 303.99603, found 303.99549.

2-Morpholino-1-(naphthalen-2-yl)-2-thioxoethan-1-one (**8**). This compound was synthesized according to General Procedure I using 2-bromo-1-(naphthalene-2-yl) ethanone (0.50 g, 2.01 mmol), morpholine (0.53 mL, 6.04 mmol), and sulfur (0.09 g, 3.01 mmol) in DMF (10 mL). Cyclohexane was used for recrystallization to afford the title compound as a yellow solid (0.31 g, 28%). R*_f_* 0.2 (Cyclohexane/EtOAc: 8/2). M.p.: 152–154 °C. ^1^H NMR (400 MHz, CDCl_3_): *δ*H (ppm) 3.63–3.65 (m, 2H), 3.69–3.71 (m, 2H), 3.93–3.96 (m, 2H), 4.38–4.41 (m, 2H), 7.55–7.66 (m, 2 ArH), 7.88–7.97 (m, 3 ArH), 8.03–8.05 (dd, 1 ArH, *J* = 2.1 and 8.8 Hz), 8.52 (s, 1 ArH). ^13^C NMR (100 MHz, CDCl_3_): *δ*C (ppm) 46.92, 51.70, 66.14, 66.25, 124.02, 126.87, 127.63, 128.67, 129.06, 129.54, 130.28, 132.11, 132.22, 135.88, 187.75 (C=O), 195.51 (C=S). HRMS (ESI^+^): *m/z* calcd for C_16_H_15_NO_2_S (M + Na)^+^ 308.07157, found 308.07146.

1-Morpholino-1-thioxobutan-2-one (**9**). This compound was synthesized according to General Procedure I using 1-bromobutan-2-one (0.34 mL, 3.33 mmol), morpholine (0.88 mL, 10.0 mmol), and sulfur (0.15 g, 4.99 mmol) in DMF (10 mL). The residue was purified by silica gel chromatography (Hexane/EtOAc: 7/3) to give the title compound as a yellow solid (0.08 g, 8%). R*_f_* 0.5 (Cyclohexane/EtOAc: 3/2). ^1^H NMR (400 MHz, CDCl_3_): *δ*H (ppm) 1.19 (t, 3H, *J* = 5.1 Hz), 2.88–2.94 (q, 2H, *J* = 5 Hz), 3.59–3.62 (m, 2H), 3.74–3.76 (m, 2H), 3.82–3.85 (m, 2H), 4.17–4.20 (m, 2H). 13C NMR (100 MHz, CDCl3): *δ*C (ppm) 7.41, 33.52, 46.94, 51.45, 65.92, 66.21, 197.42 (C+O), 199.14 (C=S) HRMS (ESI^+^): *m/z* calcd for C_8_H_14_NO_2_S [M + H]^+^ 188.07398, found 188.07384.

1-((1*r*,3*r*,5*r*,7*r*)-Adamantan-2-yl)-2-morpholino-2-thioxoethan-1-one (**10**). This compound was synthesized according to General Procedure I using 2-bromo-1-((1 r,3r,5r,7r)-Adamantan-2-yl)-10-ethanone (1 g, 3.90 mmol), morpholine (1.02 mL, 11.70 mmol), and sulfur (0.18 g, 5.90 mmol) in DMF (10 mL). The residue was purified by silica gel chromatography (cyclohexane/EtOAc: 8/2) to give the title compound as yellow oil (0.08 g, 8%). R*_f_* 0.3 (hexane/EtOAc 8:2). ^1^H NMR (400 MHz, CDCl_3_): *δ*H (ppm) 1.69–1.77 (m, 6H), 2.02–2.05 (m, 9H), 3.51 (m, 2H), 3.73–3.75 (t, 2H, *J* = 4.8 Hz), 3.80–3.83 (t, 2H, *J* = 4.8 Hz), 4.18 (m, 2H). ^13^C NMR (100 MHz, CDCl_3_): *δ*C (ppm) 27.96 (3C), 36.30 (3C), 39.79 (3C), 45.03, 46.41, 52.39, 66.30, 66.36, 196.66 (C=O), 205.54 (C=S). HRMS (ESI^+^): *m/z* calcd for C_16_H_23_NO_2_S [M + H]^+^ 294.1522, found 294.1523.

3,3-Dimethyl-1-morpholino-1-thioxobutan-2-one (**11**). This compound was synthesized according to General Procedure I using 1-bromo-3,3-dimethylbutan-2-one (2.0 g, 11.2 mmol), morpholine (2.96 mL, 33.7 mmol), and sulfur (0.54 g, 16.8 mmol) in DMF (10 mL). The residue was purified by silica gel chromatography (Cyclohexane/EtOAc: 8/2) to give the title compound as orange oil (0.35 g, 14%). R*_f_* 0.2 (Cyclohexane/EtOAc: 8/2). ^1^H NMR (400 MHz, CDCl_3_): *δ*H (ppm) 1.34 (s, 9H), 3.50–3.53 (m, 2H), 3.74–3.76 (m, 2H), 3.81–3.83 (m, 2H), 4.18 (broad, 2H). ^13^C NMR (100 MHz, CDCl_3_): *δ*C (ppm) 26.16 (3C), 40.52, 44.11, 50.03, 64.01 (2C), 194.65 (C=O), 204.21 (C=S). HRMS (ESI^+^): *m/z* calcd for C_10_H_18_NO_2_S [M + H]^+^ 216.10528, found 216.10524.

Compounds **12**–**21** were synthesized according to General Procedure II.

#### 4.1.2. General Procedure II

To commercial 2-bromo-1-phenylethanone were added, in sequence, DMF, cyclooctasulfur (1.5 equiv.), and amine derivative (3 equiv.). The mixture was then stirred at room temperature. After completion, the reaction mixture was quenched with distilled water to give a precipitate, which was further washed with distilled water. The residue was recrystallized or purified by silica gel chromatography if necessary.

1-Phenyl-2-(pyrrolidin-1-yl)-2-thioxoethan-1-one (**12**). This compound was synthesized according to General Procedure II using 2-bromo-1-phenylethanone (0.5 g, 2.52 mmol), pyrrolidine (0.62 mL, 7.56 mmol), and sulfur (0.12 g, 3.78 mmol) in DMF (10 mL). The residue was purified by silica gel chromatography (Cyclohexane/EtOAc: 8/2) to give the title compound as a yellow solid (0.12 g, 22%). R*_f_* 0.4 (Cyclohexane/EtOAc: 8/2). M.p.: 57–59 °C. ^1^H NMR (400 MHz, CDCl_3_): *δ*H (ppm) 1.61 (m, 4H), 3.53–3.56 (m, 2H), 3.94–3.98 (m, 2H), 7.46–7.48 (m, 2H), 7.50–7.62 (m, 1H), 7.99–8.01 (m, 2H). ^13^C NMR (100 MHz, CDCl_3_): *δ*C (ppm) 22.97, 25.24, 50.22, 50.40, 127.97 (2C), 129.24 (2C), 131.96, 133.37, 187.92 (C=O), 191.87 (C=S). HRMS (ESI^+^): *m/z* calcd for C_12_H_14_NOS [M + H]^+^ 220.07906, found 220.07877.

2-(4,4-Difluoropiperidin-1-yl)-1-phenyl-2-thioxoethan-1-one (**13**). This compound was synthesized according to General Procedure II using 2-bromo-1-phenylethanone (0.5 g, 2.52 mmol), 4,4-difluoropiperidine HCl (1.19 g, 7.56 mmol), and sulfur (0.12 g, 3.78 mmol) in DMF (10 mL). Cyclohexane was used for recrystallization to afford the title compound as a white solid (0.12 g, 18%). R*_f_* 0.8 (Cyclohexane/EtOAc: 3/2). M.p.: 117–119 °C. ^1^H NMR (400 MHz, CDCl_3_): *δ*H (ppm) 1.42–1.58 (m, 2H), 2.08–2.25 (m, 2H), 3.68 (m, 2H), 4.44 (m, 2H), 7.48–7.52 (m, 2H), 7.61–7.65 (m, 1H), 7.98–8.00 (m, 2H). ^13^C NMR (100 MHz, CDCl_3_): *δ*C (ppm) 31.25 (t), 32.32 (t), 41.73 (t), 46.22 (t), 118.84, and 121.26 (C-F), 127.18 (2C), 128.02 (2C), 131.23, 132.77, 186.10 (C=O), 194.87 (C=S). HRMS (ESI^+^): *m/z* calcd for C_13_H_14_NOSF_2_ [M + H]^+^ 270.07587, found 270.07568.

2-(4-Methylpiperidin-1-yl)-1-phenyl-2-thioxoethan-1-one (**14**). This compound was synthesized according to General Procedure II using 2-bromo-1-phenylethanone (1.0 g, 5.05 mmol), 4-methylpiperidine (3.82 g, 15.15 mmol), and sulfur (0.24 g, 7.57 mmol) in DMF (10 mL). The residue was purified by silica gel chromatography (Hexane/EtOAc: 9/1) to give the title compound as a yellow solid (0.47 g, 38%). R*_f_* 0.3 (Hexane/EtOAc: 7/3). M.p.: 71–73 °C. ^1^H NMR (400 MHz, CDCl_3_): *δ*H (ppm) 1.00 (s, 3H), 1.15–1.25 (m, 1H), 1.30 (m, 1H), 1.78–1.92 (m, 3H), 3.08–3.14 (m, 1H) 1.15–1.20 (m, 3H), 3.26–3.32 (m, 1H), 3.74–3.76 (m, 1H), 5.39 (m, 1H), 7.47–7.49 (m, 2H), 7.59 (m, 1H), 7.98 (m, 2H). ^13^C NMR (100 MHz, CDCl_3_): *δ*C (ppm) 18.86, 28.42, 30.95, 31.97, 45.03, 49.83, 126.48 (2C), 127.41 (2C), 131.01, 131.82, 185.65 (C=O), 192.08 (C=S). HRMS (ESI^+^): *m/z* calcd for C_14_H_17_NOSNa (M + Na)^+^ 270.09231, found 270.09219.

1-Phenyl-2-(piperidin-1-yl)-2-thioxoethan-1-one (**15**). This compound was synthesized according to General Procedure II using 2-bromo-1-phenylethanone (2.0 g, 10.01 mmol), piperidine (2.99 mL, 30.30 mmol), and sulfur (0.48 g, 15.10 mmol) in DMF (10 mL). The residue was purified by silica gel chromatography (Cyclohexane/EtOAc: 8/2) to give the title compound as a yellow solid (0.47 g, 20%). R*_f_* 0.6 (Cyclohexane/EtOAc: 3/2). M.p.: 67–69 °C. ^1^H NMR (400 MHz, CDCl_3_): *δ*H (ppm) 1.56–1.63 (m, 2H), 1.77–1.84 (m, 4H), 3.53–3.55 (m, 2H), 4.24–4.27 (m, 2H), 7.47–7.50 (m, 2H), 7.58–7.62 (m, 1H), 7.98–8.00 (m, 2H). ^13^C NMR (100 MHz, CDCl_3_): *δ*C (ppm) 23.79, 25.13, 26.20, 47.88, 52.79, 52.79, 128.63 (2C), 129.52 (2C), 133.14, 133.97, 187.76 (C=O), 194.03 (C=S). 18.86, 28.42, 30.95, 31.97, 45.03, 49.83, 126.48 (2C), 127.41 (2C), 131.01, 131.82, 185.65 (C=O), 192.08 (C=S). HRMS (ESI^+^): *m/z* calcd for C_13_H_15_NOSNa (M + Na)^+^ 256.07666, found 256.07661.

*N*,*N*-Diethyl-2-oxo-2-phenylethanethioamide (**16**). This compound was synthesized according to General Procedure II using 2-bromo-1-phenylethanone (1.0 g, 5.05 mmol), diethylamine (1.56 mL, 15.15 mmol), and sulfur (0.24 g, 7.57 mmol) in DMF (10 mL). The residue was purified by silica gel chromatography (Hexane/EtOAc: 7/3) to give the title compound as brown oil (0.09 g, 8%). R*_f_* 0.3 (Hexane/EtOAc: 7/3).^1^H NMR (400 MHz, CDCl_3_): *δ*H (ppm) 1.15–1.20 (m, 3H), 1.26–1.34 (m, 3H), 3.37–3.42 (m, 2H), 3.94–3.99 (m, 2H), 7.36–7.40 (m, 2H), 7.48–7.52 (m, 1H), 7.87–7.89 (m, 2H). ^13^C NMR (100 MHz, CDCl_3_): *δ*C (ppm) 11.29, 13.63, 44.55, 47.99, 128.80 (2C), 129.90 (2C), 133.51, 134.09, 187.51 (C=O), 195.58 (C=S). HRMS (ESI^+^): *m/z* calcd for C_12_H_16_NOS [M + H]^+^ 222.09471, found 222.09456.

1-Phenyl-2-thiomorpholino-2-thioxoethan-1-one (**17**). This compound was synthesized according to General Procedure II using 2-bromo-1-phenylethanone (1.0 g, 5.05 mmol), thiomorpholine (1.52 mL, 15.15 mmol), and sulfur (0.24 g, 7.57 mmol) in DMF (10 mL). The residue was purified by silica gel chromatography (Hexane/EtOAc: 7/3) to give the title compound as a yellow solid (0.40 g, 43%). R*_f_* 0.7 (Cyclohexane/EtOAc: 3/2). M.p.: 127–129 °C. ^1^H NMR (400 MHz, CDCl_3_): *δ*H (ppm) 2.69 (m, 2H), 2.88–2.90 (t, 2H, *J* = 5.1 Hz), 3.83–3.86 (t, 2H, *J* = 5.1 Hz), 4.59 (m, 2H), 7.47–7.51 (m, 2H), 7.59–7.63 (m, 1H), 7.97–7.99 (m, 2H). ^13^C NMR (100 MHz, CDCl_3_): *δ*C (ppm) 27.31, 28.25, 49.71, 54.64, 128.96 (2C), 129.90 (2C), 133.23, 134.44, 187.73 (C=O), 196.06 (C=S). HRMS (ESI^+^): *m/z* calcd for C_12_H_14_NOS_2_ [M + H]^+^ 252.05113, found 252.05117.

2-(4-Methylpiperazin-1-yl)-1-phenyl-2-thioxoethan-1-one (**18**). This compound was synthesized according to General Procedure II using 2-bromo-1-phenylethanone (2 g, 10.10 mmol), *N*-methylpiperazine (3.37 mL, 30.30 mmol), and sulfur (0.48 g, 15.15 mmol) in DMF (10 mL). The residue was purified by silica gel chromatography (Cyclohexane/EtOAc: 8/2) to give the title compound as brown solid (0.58 g, 23%). R*_f_* 0.2 (Cyclohexane/EtOAc: 8/2). M.p.: 93–95 °C ^1^H NMR (400 MHz, CDCl_3_): *δ*H (ppm) 2.34 (s, 3H, CH3), 2.41–2.44 (m, 2H), 2.62–2.64 (m, 2H), 3.58–3.60 (m, 2H), 4.31–4.34 (m, 2H), 7.47–7.51 (m, 2H), 7.59–7.63 (m, 1H), 7.98–8.00 (m, 2H). ^13^C NMR (100 MHz, CDCl_3_): *δ*C (ppm) 45.53, 46.61, 51.31, 53.95, 54.57, 128.78 (2C), 129.71 (2C), 133.18, 134.21, 187.80 (C=O), 195.17 (C=S). HRMS (ESI^+^): *m/z* calcd for C_13_H_17_N_2_OS [M + H]^+^ 249.10561, found 249.10561.

2-(4-(2-Oxo-2-phenylethyl)piperazin-1-yl)-1-phenyl-2-thioxoethan-1-one (**19**). This compound was synthesized according to General Procedure I using 2-bromo-1-phenylethanone (1.00 g, 5.05 mmol), piperazine (1.30 g, 15.15 mmol), and sulfur (0.24 g, 7.57 mmol) in DMF (10 mL). The residue was purified by silica gel chromatography (hexane/EtOAc: 9/1) to give the title compound as orange oil (0.40 g, 22%). R*_f_* 0.2 (cyclohexane/EtOAc: 8/2). ^1^H NMR (400 MHz, CDCl_3_): *δ*H (ppm) 2.67–2.70 (t, 2H, *J* = 4.8 Hz), 2.87–2.90 (t, 2H, *J* = 4.8 Hz), 3.66–3.69 (t, 2H, *J* = 4.8 Hz), 4.40–4.43 (t, 2H, *J* = 4.8 Hz), 7.42–7.53 (m, 4 ArH), 7.58–7.64 (m, 2 ArH), 7.94–8.15 (m, 4 ArH). HRMS (ESI^+^): *m/z* calcd for C_20_H_20_N_2_O_2_S [M + H]^+^ 353.1318, found 353.1318.

1-Phenyl-2-(4-phenylpiperazin-1-yl)-2-thioxoethan-1-one (**20**). This compound was synthesized according to General Procedure II using 2-bromo-1-phenylethanone (1.0 g, 5.05 mmol), 1-phenyl-piperazine (2.31 mL, 15.15 mmol), and sulfur (0.24 g, 7.57 mmol) in DMF (10 mL). The residue was purified by silica gel chromatography (Cyclohexane/EtOAc: 8/2) to give the title compound as a yellow solid (0.20 g, 13%). R*_f_* 0.4 (Cyclohexane/EtOAc: 8/2). M.p.: 89–91 °C. ^1^H NMR (400 MHz, CDCl_3_): *δ*H (ppm) 3.20–3.22 (m, 2H), 3.40–3.43 (m, 2H), 3.73–3.76 (m, 2H), 4.46–4.48 (m, 2H), 6.92–6.96 (m, 3H), 7.28–7.31 (m, 2H), 7.48–7.52 (m, 2H), 7.60–7.64 (m, 1H), 8.00–8.02 (m, 2H). ^13^C NMR (100 MHz, CDCl_3_): *δ*C (ppm) 45.06, 47.45, 48.10, 49.66, 115.28 (2C), 119.55, 126.78, 127.30 (2C), 127.74 (2C), 128.24 (2C), 131.65, 132.77, 148.47, 186.30 (C=O), 193.88 (C=S). HRMS (ESI^+^): *m/z* calcd for C_18_H_19_N_2_OS [M + H]^+^ 311.12181, found 311.12068.

2-(4-Benzhydrylpiperazin-1-yl)-1-phenyl-2-thioxoethan-1-one (**21**). This compound was synthesized according to General Procedure II using 2-bromo-1-phenylethanone (1.0 g, 5.05 mmol), 1-(diphenylmethyl)piperazine (3.82 g, 15.15 mmol) and sulfur (0.24 g, 7.57 mmol) in DMF (10 mL). Cyclohexane was used for recrystallization to afford the title compound as a white solid (0.60 g, 30%). R_f_ 0.5 (Hexane/EtOAc: 8/2). M.p.: 130–132 °C. ^1^H NMR (400 MHz, CDCl_3_): *δ*H (ppm) 2.40 (m, 2H), 2.62 (m, 2H), 3.57 (m, 2H), 4.29 (m, 3H), 7.17–7.25 (m, 6H), 7.39–7.47 (m, 6H), 7.56–7.59 (m, 1H), 7.95–7.96 (m, 2H). ^13^C NMR (100 MHz, CDCl_3_): *δ*C (ppm) 45.65, 49.77, 50.33, 50.41, 74.22, 126.09 (2C), 126.41 (4C), 127.44 (4C), 127.55 (2C), 128.48 (2C), 131.99, 132.94, 140.26 (2C), 186.66 (C=O), 193.69 (C=S). HRMS (ESI^+^): *m/z* calcd for C_25_H_25_N_2_OS [M + H]^+^ 401.16821, found 401.16826.

#### 4.1.3. Synthesis of Compounds **22**–**39**

Morpholino(phenyl)methanone (**22**). To a stirring solution of benzoic acid (2.50 g, 20.47 mmol, 1 equiv.) in CH_2_Cl_2_ (50 mL) were added catalytic amount of DMF and oxalyl chloride (2.60 g, 20.47 mmol, 1 equiv.). The reaction mixture was stirred at room temperature until the generation of gasses was stopped (HCl), cooled to 0 °C, and a solution of morpholine (2.67 g, 30.7 mmol, 1.5 equiv.) and DIPEA (5.29 g, 40.94 mmol, 2 equiv.) was slowly added. The mixture was stirred at room temperature for 6 h. The reaction was quenched by adding water (50 mL) following by extraction with CH_2_Cl_2_ (75 mL). The combined organic layer were washed with brine and dried over Na_2_SO_4_. The solvent was removed in vacuo, and the residue was purified by silica gel chromatography (Cyclohexane/EtOAc: 2/3) to give the title compound as a white solid (17.81 mmol, 3.40 g, 87%). R*_f_* 0.45 (Cyclohexane/EtOAc: 3/2). M.p.: 62–63 °C. ^1^H NMR (400 MHz, CDCl_3_): *δ*H (ppm) 3.41–3.50 (m, 4H), 3.64–3.79 (m, 4H), 7.39–7.44 (m, 5H). ^13^C NMR (100 MHz, CDCl_3_): *δ*C (ppm) 26.84, 42.60, 48.19, 66.74, 127.20 (2C), 128.67 (2C), 129.99, 135.24, 170.32 (C=O). HRMS (ESI^+^): *m/z* calcd for C_11_H_14_NO_2_ [M + H]^+^ 192.1024, found 192.1014.

Morpholino(phenyl)methanethione (**23**). Benzaldehyde (1.00 g, 9.42 mmol, 1 equiv.), morpholine (2.05 g, 23.5 mmol, 2.5 equiv.), sulfur (0.30 g, 9.42 mmol, 1 equiv.), and *para*-toluene sulfonic acid (0.05 g, 0.26 mmol, 0.028 equiv.) were mixed together and the solution stirred at 85 °C for 12 h. The brown solid formed was recovered with 50 mL of CH_2_Cl_2_. The solution was then filtered and the organic layer was extracted 3 times with water (80 mL). The organic layer was evaporated under vacuo and the desired product was recrystallized in EtOH to give the title compound as a yellow solid (5.84 mmol, 1.21 g, 62%). R*_f_* 0.7 (Cyclohexane/EtOAc: 2/3). M.p.: 128–129 °C. ^1^H NMR (400 MHz, CDCl_3_): *δ*H (ppm) 3.59–3.62 (m, 2H), 3.64–3.66 (m, 2H), 3.88–3.91 (m, 2H), 4.44–4.46 (m, 2H), 7.27–7.30 (m, 2H), 7.34–7.39 (m, 3H). ^13^C NMR (100 MHz, CDCl_3_): *δ*C (ppm) 47.32, 50.29, 64.33, 64.54, 123.57 (2C), 126.45 (2C), 126.68, 140.13, 198.90 (C=S). HRMS (ESI^+^): *m/z* calcd for C_11_H_14_NOS [M + H]^+^ 208.0796, found 208.0791.

1-Morpholino-2-phenylethane-1,2-dione (**24**). To a solution of 2-oxo-2-phenylacetic acid (2.50 g, 16.6 mmol, 1 equiv.) in CH_2_Cl_2_ were added catalytic amount of DMF and oxalyl chloride (2.11 g, 16.6 mmol, 1 equiv.). The reaction was stirred at room temperature until the generation of gasses (HCl) was stopped, cooled to 0 °C and a solution of morpholine (2.17 g, 24.9 mmol, 1.5 equiv.) with DIPEA (4.29 g, 33.2 mmol, 2 equiv.) was slowly added. The mixture was stirred at room temperature for 14 h, quenched by adding water and extracted with CH_2_Cl_2_. The combined organic layer were washed with brine and dried over Na_2_SO_4_. The solvents were removed in vacuo, and the resulting crude product was purified by silica gel chromatography (Cyclohexane/EtOAc: 3/2) to give the title compound as a white solid (16.4 mmol, 3.60 g, 99%). R*_f_* 0.35 (Cyclohexane/EtOAc: 3/2). M.p.: 50–52 °C. ^1^H NMR (400 MHz, CDCl_3_): *δ*H (ppm) 3.38–3.40 (m, 2H), 3.65–3.68, (m, 2H), 3.80 (m, 4H), 7.51–7.55 (m, 2H), 7.65–7.69 (m, 1H), 7.96–7.98 (m, 2H). ^13^C NMR (100 MHz, CDCl_3_): *δ*C (ppm) 41.64, 46.28, 66.70, 66.77, 129.14 (2C), 129.71 (2C), 133.06, 134.99, 165.48 (C=O), 191.19 (C=O). HRMS (ESI^+^): *m/z* calcd for C_12_H_14_NO_3_ [M + H]^+^ 220.0973, found 220.0968.

1-Morpholino-2-phenylethane-1-thione (**25**). To a solution of acetophenone (1.50 g, 12.48 mmol, 1 equiv.) and sulfur (0.60 g, 18.73 mmol, 1.5 equiv.) in DMF (20 mL), morpholine (3.26 g, 12.48 mmol, 3 equiv.) was slowly added and the reaction was stirred at reflux for 10 h. After the completion of the reaction, water was added on the mixture and the solution was extracted with EtOAc (150 mL). The organic layers were dried and evaporated under vacuo to afford the crude mixture. The residue was purified by silica gel chromatography (Cyclohexane/EtOAc: 2/3) to give the title compound as a yellow solid (4.87 mmol, 1.08 g, 39%). R*_f_* 0.67 (EtOAc/Cyclohexane: 3/2). M.p.: 66–67 °C. ^1^H NMR (400 MHz, (CD_3_)_2_SO): *δ*H (ppm) 3.34 (s, 2H), 3.40–3.42 (m, 2H), 3.63–3.66 (m, 2H), 3.69–3.72 (m, 2H), 4.22–4.25 (m, 2H), 7.23–7.28 (m, 1H), 7.32–7.35 (m, 4H). ^13^C NMR (100 MHz, (CD_3_)_2_SO): *δ*C (ppm) 48.66, 49.40, 50.22, 65.23, 65.33, 126.27, 127.53 (2C), 128.19 (2C), 135.95, 198.39 (C=S). HRMS (ESI^+^): *m/z* calcd for C_12_H_16_NOS [M + H]^+^ 222.0952, found 222.0941.

2-Morpholino-1-phenylethan-1-one (**26**). 2-bromo-1-phenylethanone (1.00 g, 5.02 mmol, 1 equiv.) and morpholine (1.71 g, 19.59 mmol, 3.9 equiv.) were mixed in a solution of K_2_CO_3_ (3.12 g, 22.59 mmol, 4.5 equiv.) in acetonitrile and stirred for 3 h. Then the solution was filtered to remove K_2_CO_3_ and evaporated under reduced pressure. The resulting crude product was purified by silica gel chromatography (Cyclohexane/EtOAc: 3/2) to give the title compound as red oil (1.71 mmol, 0.35 g, 34%). R*_f_* 0.4 (Cyclohexane/EtOAc: 3/2). ^1^H NMR (400 MHz, CDCl_3_): *δ*H (ppm) 261–2.63 (m, 4H), 3.78–3.80 (m, 4H), 3.83 (s, 2H), 7.45–7.49 (m, 2H), 7.56–7.60 (m, 1H), 7.99–8.01 (m, 2H). ^13^C NMR (100 MHz, CDCl_3_): *δ*C (ppm) 53.91 (2C), 64.72, 66.84 (2C), 128.09 (2C), 128.61 (2C), 133.37, 135.95, 196.10 (C=O). HRMS (ESI^+^): *m/z* calcd for C_11_H_16_NO_2_ [M + H]^+^ 206.11810, found 206.11425. Data are in agreement with the literature [12].

2-Hydroxy-1-morpholino-2-phenylethan-1-one (**27**). To a solution of 1-morpholino-2-phenylethane-1,2-dione (0.25 g, 1.14 mmol, 1 equiv.) in 10 mL of MeOH, NaBH_4_ (0.11 g, 2.85 mmol, 2.5 equiv.) was added and stirred during 5 h. The reaction mixture was then evaporated, diluted in water, and extracted with CH_2_Cl_2_. The organic layer was evaporated and purified by silica gel chromatography (Cyclohexane/EtOAc: 8/2) to give the title compound as a white solid (0.59 mmol, 0.13 g, 52%). R*_f_* 0.53 (Cyclohexane/EtOAc: 8/2). M.p.: 85–87 °C. ^1^H NMR (400 MHz, CDCl_3_): *δ*H (ppm) 3.02–3.09 (m, 1H), 3.13–3.18 (m, 1H), 3.44–3.49 (m, 1H), 3.52–3.63 (m, 2H), 3.67–3.71 (m, 1H), 3.76–3.81 (m, 1H), 4.73 (s, 1H, OH), 5.19 (s, 1H), 7.29–7.40 (m, 5H). ^13^C NMR (100 MHz, CDCl_3_): *δ*C (ppm) 43.15, 45.31, 65.81, 66.56, 71.54, 127.39 (2C), 128.74, 129.21 (2C), 139.13, 171.03 (C=O). HRMS (ESI^+^): *m/z* calcd for C_12_H_16_NO_3_ [M + H]^+^ 222.1130, found 222.1125.

1-Morpholino-2-phenylethan-1-one (**28**). To a solution of 2-phenylacetic-acid (0.50 g, 3.70 mmol, 1 equiv.) in CH_2_Cl_2_ were added catalytic amounts of DMF and oxalyl chloride (0.47 g, 3.7 mmol, 1 equiv.). The reaction was stirred at room temperature until the generation of gasses (HCl) was stopped. The resulting solution was cooled to 0 °C and a solution of morpholine (0.48 g, 5.55 mmol, 1.5 equiv.) and DIPEA (0.96 g, 7.40 mmol, 2 equiv.) was slowly added. The mixture was stirred at room temperature for 14 h, quenched by adding water (100 mL), and the mixture was extracted with CH_2_Cl_2_ (150 mL). The combined organic layers were washed with brine and dried over Na_2_SO_4_. The residue was purified by silica gel chromatography (Cyclohexane/EtOAc: 2/3) to give the title compound as a white solid (2.90 mmol, 0.59 g, 78%). R*_f_* 0.29 (Cyclohexane/EtOAc: 2/3). M.p.: 60–62 °C. ^1^H NMR (400 MHz, CDCl_3_): *δ*H (ppm) 3.42–3.45 (m, 2H), 3.47–3.49 (m, 2H), 3.65 (s, 4H), 3.74 (s, 2H), 7.23–7.27 (m, 3H), 7.31–7.35 (m, 2H). ^13^C NMR (100 MHz, CDCl_3_): *δ*C (ppm) 39.10, 40.36, 44.75, 64.69, 65.03, 125.23, 126.75 (2C), 127.06 (2C), 133.02. HRMS (ESI^+^): *m/z* calcd for C_12_H_16_NO_2_ [M + H]^+^ 206.1181, found 206.1176.

*N*-Phenylmorpholine-4-carboxamide (**29**). To a MeCN solution of ethyl morpholine-4-carboxylate (0.40 g, 2.50 mmol, 1 equiv.) were added POCl_3_ (1.15 g, 7.50 mmol, 3 equiv.) under Ar. The reaction mixture was stirred at reflux and monitored by TLC. After completion, the solution was diluted in water and extracted with CH_2_Cl_2_. The organic layer was evaporated, diluted in dry CH_2_Cl_2_, cooled to 0 °C, and a solution of aniline (0.35 g, 3.75 mmol, 1.5 equiv.) and DIPEA (0.65 g, 3.75 mmol, 2 equiv.) in CH_2_Cl_2_ was slowly added. The reaction mixture was stirred at room temperature for 50 h. The reaction was quenched by adding water and the mixture was extracted with CH_2_Cl_2_. The combined organic layers were washed with brine and dried over Na_2_SO_4_. The resulting crude product was purified by silica gel chromatography (Cyclohexane/EtOAc: 8/2) to give the title compound as a green solid (1.32 mmol, 0.27 g, 53%). R*_f_* 0.14 (Cyclohexane/EtOAc: 3/2). M.p.: 156–158 °C. ^1^H NMR (400 MHz, CDCl_3_): *δ*H (ppm) 3.48–3.50 (m, 4H), 3.74–3.77 (m, 4H), 6.69 (s, 1H, NH), 7.04–7.08 (m, 1H), 7.28–7.37 (m, 4H). ^13^C NMR (100 MHz, CDCl_3_): *δ*C (ppm) 44.26 (2C), 66.51 (2C), 120.14 (2C), 123.40, 128.95 (2C), 138.71, 155.19 (C=O). HRMS (ESI^+^): *m/z* calcd for C_11_H_15_N_2_O_2_ [M + H]^+^ 207.1133, found 207.1128.

*N*-Morpholinobenzamide (**30**). To a solution of benzoic acid (2.50 g, 20.47 mmol, 1 equiv.) in CH_2_Cl_2_, a catalytic amount of DMF and oxalyl chloride were added (2.60 g, 20.47 mmol, 1 equiv.) and the mixture was stirred at room temperature until the generation of gasses (HCl) was stopped. The reaction was cooled to 0 °C before adding a solution of morpholin-4-amine (3.13 g, 30.7 mmol, 1.5 equiv.) and DIPEA (4.53 g, 40.94 mmol, 2 equiv.) and the reaction was stirred at room temperature for 6 h. The solution was quenched by water (30 mL) and the mixture was extracted with CH_2_Cl_2_ (100 mL). The combined organic layers were washed with brine and dried over Na_2_SO_4_. The solvent was removed in vacuo, and the resulting crude product was purified by silica gel chromatography (Cyclohexane/EtOAc: 3/2) to give the title compound as yellow oil (2.05 mmol, 0.42 g, 10%). R*_f_* 0.25 (Cycloxane/EtOAc: 3/2). ^1^H NMR (400 MHz, CDCl_3_): *δ*H (ppm) 2.96–2.97 (m, 4H), 3.87–3.89 (m, 4H), 6.84 (s, 1H), 7.40–7.56 (m, 2H), 7.52–7.54 (m, 1H), 7.74–7.75 (m, 2H). ^13^C NMR (100 MHz, CDCl_3_): *δ*C (ppm) 56.12 (2C), 66.44 (2C), 127.05, 128.69 (2C), 128.69 (2C), 144.76, 151.61(C=O). HRMS (ESI^+^): *m/z* calcd for C_11_H_15_N_2_O_2_ [M + H]^+^ 207.11335, found 207.11221.

(*E*)-*N*-Morpholino-1-phenylmethanimine (**31**). To a solution of MgSO_4_ (2.27 g, 18.86 mmol, 2 equiv.) in CH_2_Cl_2_ was added benzaldehyde (0.96 mL, 9.43 mmol, 1 equiv.) under Ar and the solution was stirred overnight at room temperature. The solution was filtered to remove MgSO_4_ and evaporated under reduced pressure. The resulting crude product was purified by silica gel chromatography (CH_2_Cl_2_) to give the title compound as a white solid (7.35 mmol, 1.39 g, 78%). R*_f_* 0.2 (CH_2_Cl_2_). M.p.: 86–88 °C. ^1^H NMR (400 MHz, CDCl_3_): *δ*H (ppm) 3.17–319 (m, 4H), 3.88–3.91 (m, 4H), 7.26–730 (m, 1H), 7.33–7.37 (m, 2H), 7.60–7.61 (m, 3H). ^13^C NMR (100 MHz, CDCl_3_): *δ*C (ppm) 51.88 (2C), 66.50 (2C), 126.23 (2C), 128.40, 128.58 (2C), 135.92, 136.31(C=N). HRMS (ESI^+^): *m/z* calcd for C_11_H_15_N_2_O [M + H]^+^ 191.11844, found 191.11733.

1-Morpholino-3-phenylthiourea (**32**). To a solution of isothiocyanatobenzene (1.00 g, 7.40 mmol, 1 equiv.) in MeOH, 4-amino-morpholin (0.91 g, 8.9 mmol, 1.2 equiv.) was slowly added and the reaction was monitored with TLC until complete disappearance of the starting material and the formation of a white solid. The solution was then filtered and residue recovered was recrystallized in MeOH to afford the desired product as a white solid (5.92 mmol, 1.40 g, 80%). R*_f_* 0.35 (Cyclohexane/EtOAc: 3/2). M.p.: 160–161 °C. ^1^H NMR (400 MHz, CDCl_3_): *δ*H (ppm) 2.99–3.02 (m, 4H), 3.74–3.76 (m, 4H), 7.54–7.59 (m, 2H) 7.62–7.66 (m, 1H), 7.75–7.78 (m, 2H). ^13^C NMR (100 MHz, CDCl_3_): *δ*C (ppm) 45.54 (2C), 65.74 (2C), 127.45 (2C), 128.80 (2C), 132.64, 134.60, 157.40 (C=S). HRMS (ESI^+^): *m/z* calcd for C_11_H_16_N_3_OS [M + H]^+^ 238.1114, found 238.1009.

1-Morpholino-3-phenylurea (**33**). To a stirring solution of phenylisocyanate (1.00 g, 8.4 mmol, 1 equiv.) in CH_2_Cl_2_, 4-amino-morpholin (1.03 g, 10.1 mmol, 1.2 equiv.) was slowly added and the reaction was monitored with TLC until complete disappearance of the starting material. A white solid appeared in the mixture. The solution was filtered and the residue was purified by silica gel chromatography (EtOAc) to give the title compound as a white solid (6.64 mmol, 1.47 g, 79%). R*_f_* 0.4 (EtOAc). M.p.: 147–148 °C. ^1^H NMR (400 MHz, CDCl_3_): *δ*H (ppm) 2.67–3.05 (m, 4H), 3.71–3.91 (m, 4H), 5.53 (s, 1H), 7.04–7.08 (m, 1H), 7.29–7.33 (m, 2H), 7.47–7.49 (m, 2H), 8.05 (s, 1H). ^13^C NMR (100 MHz, CDCl_3_): *δ*C (ppm) 56.81 (2C), 66.75 (2C), 119.21 (2C), 123.26, 129.02 (2C), 138.07, 154.75 (C=O). HRMS (ESI^+^): *m/z* calcd for C_11_H_14_NO_2_ [M + H]^+^ 222.1242, found 222.1231.

Benzyl morpholine-4-carbodithioate (**34**). To a solution of CS_2_ (0.5 g, 6.6 mmol, 1 equiv.) in diethyl ether, morpholine (1.13 mL, 13.2 mmol, 2 equiv.) was slowly added. The resulting mixture was stirred for 1 h at room temperature. The precipitated salt was recovered by filtration and dried over vacuum. The morpholinium salt (0.5 g, 2.0 mmol, 1 equiv.) was then mixed with (thiocyanatomethyl)benzene (0.3 g, 2.0 mmol, 1 equiv.) in acetonitrile and stirred at room temperature during 2 h. The solution was evaporated under vacuum, the resulting powder was diluted in 15 mL of water and extracted with EtOAc. The combined organic layers were washed with brine and dried over Na_2_SO_4_. The solvents were removed in vacuo, and the resulting crude product was purified by silica gel chromatography (Cyclohexane/EtOAc: 3/2) to give the title compound as a white solid (0.8 mmol, 0.2 g, 42%). R*_f_* 0.7 (Cyclohexane/EtOAc: 3/2). M.p.: 70–71 °C. ^1^H NMR (400 MHz, CDCl_3_): *δ*H (ppm) 3.38–3.40 (m, 2H), 3.65–3.68, (m, 2H), 3.80 (m, 4H), 3.76 (bs, 4H), 3.93–4.34 (m, 4H), 4.58 (s, 2H), 7.26–7.34 (m, 3H), 7.38–7.39 (m, 2H). ^13^C NMR (100 MHz, CDCl_3_): *δ*C (ppm) 42.03 (2C), 66.43 (2C), 127.66, 128.66 (2C), 129.41 (2C), 135.69, 197.20 (C=S). HRMS (ESI^+^): *m/z* calcd for C_12_H_16_NOS_2_ [M + H]^+^ 254.06733, found 254.06602.

(*S*)-*N*-(2-Hydroxy-1-phenylethyl)morpholine-4-carboxamide (**35**). To a solution of (*S*)-(+)-2phenylglycinol (1.00 g, 7.29 mmol, 1.5 equiv.) in anhydrous CH_2_Cl_2_ under Ar, DIPEA (1.71 mL, 9.71 mmol, 2 equiv.) and 4-morpholinylcarbonyl chloride (0.57 mL, 4.86 mmol, 1 equiv.) were added at 0 °C. The resulting solution was stirred at room temperature for 12 h. The solvent was removed in vacuo, and the resulting crude product was purified by silica gel chromatography (CH_2_Cl_2_/MeOH: 9/1) to give the title compound as a yellow solid (4.32 mmol, 1.62 g, 89%). R*_f_* 0.2 (EtOAc). M.p.: 90–91 °C. ^1^H NMR (400 MHz, CDCl_3_): *δ*H (ppm) 3.10–3.21 (m, 4H), 3.45–3.54 (m, 4H), 3.57–3.67 (m, 2H), 4.31 (s, 1H), 4.76–4.81 (m, 1H), 5.83 (d, 1H, *J* = 6.2 Hz), 7.20–7.25 (m, 3H), 7.29–7.32 (m, 2H). ^13^C NMR (100 MHz, CDCl_3_): *δ*C (ppm) 43.89 (2C), 57.11, 66.02, 66.38 (2C), 126.58 (2C), 127.43, 128.54 (2C), 140.67, 158.06 (C=O). HRMS (ESI^+^): *m/z* calcd for C_13_H_19_N_2_O_3_ [M + H]^+^ 251.13957, found 251.13835.

4-(Phenylsulfonyl)morpholine (**36**). To a solution of benzenesulfonyl chloride (1.65 g, 9.60 mmol, 1.2 equiv.) in CH_2_Cl_2_, morpholine (1.00 g, 11.5 mmol, 1 equiv.) and Et_3_N (0.97 mL, 9.60 mmol, 1.2 equiv.) were added and the reaction was stirred during 3 h. A white solid slowly appeared in the mixture. The residue was filtered and recrystallized in EtOH to afford the desired compound as a white solid (8.16 mmol, 1.85 g, 85%). R*_f_* 0.27 (Cyclohexane/EtOAc: 3/2). M.p.: 107–109 °C. ^1^H NMR (400 MHz, CDCl_3_): *δ*H (ppm) 2.99–3.02 (m, 4H), 3.74–3.76 (m, 4H), 7.54–7.59 (m, 2H), 7.62–7.66 (m, 1H), 7.75–7.78 (m, 2H). ^13^C NMR (100 MHz, CDCl_3_): *δ*C (ppm) 45.63 (2C), 65.74 (2C), 127.50 (2C), 128.80 (2C), 132.75, 134.70. HRMS (ESI^+^): *m/z* calcd for C_10_H_14_NO_3_S [M + H]^+^ 228.0694, found 228.0688.

*N*-Morpholinobenzenesulfonamide (**37**). To a solution of benzenesulfonyl chloride (1.65 g, 9.60 mmol, 1.2 equiv.) in CH_2_Cl_2_ was added 4-amino-morpholine (1.17 g, 11.5 mmol, 1 equiv.) and Et_3_N (0.97 mL, 9.60 mmol, 1.2 equiv.). The reaction was stirred for 3 h and followed by TLC. After complete disappearance of the starting material, 100 mL of water was added on the mixture and the solution was extracted with CH_2_Cl_2_ (75 mL). The organic layers were dried and evaporated under vacuum. The solid recovered was recrystallized in EtOH to afford the desired compound as a white solid (7.97 mmol, 1.93 g, 83%). R*_f_* 0.29 (Cyclohexane/EtOAc: 3/2). M.p.: 110–112 °C. ^1^H NMR (400 MHz, CDCl_3_): *δ*H (ppm) 2.60–2.63 (m, 4H), 3.60–3.62 (m, 4H), 5.34 (s, 1H, NH), 7.51–7.55 (m, 2H), 7.60–7.64 (m, 1H), 7.97–7.99 (m, 2H). ^13^C NMR (100 MHz, CDCl_3_): *δ*C (ppm) 56.74 (2C), 66.64 (2C), 128.16 (2C), 128.89 (2C), 133.21, 138.61. HRMS (ESI^+^): *m/z* calcd for C_10_H_15_N_2_O_3_S [M + H]^+^ 243.0803, found 243.0798.

(4-Chlorophenyl)(morpholino)methanethione (**38**). 4-chloro-benzaldehyde (1.00 g, 7.10 mmol, 1 equiv.), morpholine (1.86 g, 21.30 mmol, 3.0 equiv.), and sulfur (0.34 g, 10.70 mmol, 1 equiv.) were mixed in DMF and the solution stirred at 85 °C for 12 h. The solution was filtered, H_2_O (100 mL) was added and the organic layer was extracted 3 times with EtOAc. The organic layer was evaporated under vacuo, the residue was purified by silica gel chromatography (Cyclohexane/EtOAc: 4/1) to give a yellow powder. This powder was recrystallized in EtOH to give the title compound as a yellow solid (4.59 mmol, 1.11 g, 65%). R*_f_* 0.5 (Cyclohexane/EtOAc: 3/2). M.p.: 140–142 °C. ^1^H NMR (400 MHz, CDCl_3_): *δ*H (ppm) 3.60–3.65 (m, 4H), 3.87–3.89 (m, 2H), 4.41–4.43 (m, 2H), 7.23–7.25 (m, 2H), 7.33–7.35 (m, 2H). ^13^C NMR (100 MHz, CDCl_3_): *δ*C (ppm) 49.55, 52.53, 66.46, 66.62, 126.97 (2C), 128.95 (2C), 134.93, 140.60, 199.54 (C=S). HRMS (ESI^+^): *m/z* calcd for C_11_H_13_ClNOS [M + H]^+^ 242.04064, found 242.03942.

4-Chloro-*N*-morpholinobenzenesulfonamide (**39**). To a solution of 4-chlorobenzenesulfonyl chloride (1.24 g, 5.87 mmol, 1.2 equiv.) in CH_2_Cl_2_ was added 4-amino-morpholine (0.5 g, 4.89 mmol, 1 equiv.) and Et_3_N (0.82 mL, 5.87 mmol, 1.2 equiv.). The reaction was stirred for 12 h. After complete disappearance of the starting material, 100 mL of water was added on the mixture and the solution was extracted with CH_2_Cl_2_ (75 mL). The organic layers were dried and evaporated under vacuum. The solid recovered was recrystallized in EtOH to afford the desired compound as a white solid (4.34 mmol, 1.20 g, 74%). R*_f_* 0.55 (Cyclohexane/EtOAc: 3/2). M.p.: 161–163 °C. ^1^H NMR (400 MHz, CDCl_3_): *δ*H (ppm) 2.64–2.66 (m, 4H), 3.61–3.64 (m, 4H), 5.34 (s, 1H, NH), 7.50–7.52 (m, 2H), 7.90–7.92 (m, 2H). ^13^C NMR (100 MHz, CDCl_3_): *δ*C (ppm) 54.65 (2C), 64.46 (2C), 127.04 (2C), 127.44 (2C), 134.95, 137.64. HRMS (ESI^+^): *m/z* calcd for C_10_H_15_NO_3_S [M + H]^+^ 277.0414, found 277.0408.

### 4.2. WT PHGDH and PSAT-1 Purification

pET28a human PHGDH and pET28a human PSAT-1 were transformed into BL21 *Escherichia coli*. A single colony was grown to an OD_600_ 0.6 in 1 L of Terrific broth. Protein expression was induced with 1 mM isopropyl thiogalactopyranoside (IPTG). The culture was chilled on ice for 30 min, cultured for 18 h at 24 °C, and pelleted (6000× *g*, 20 min). Pellets were resuspended in 60 mL of lysis buffer (50 mM Tris, pH 8.5, 10 mM MgCl_2_, 300 mM NaCl, 10% glycerol, 5 mM imidazole) and sonicated, and cell debris were pelleted by centrifugation (20,000× *g*, 30 min). The supernatant was collected and purified using Akta purifier on HisTrap^TM^ FF column (GE Healthcare). After column equilibration with wash buffer (50 mM Tris, pH 8.5, 10 mM MgCl_2_, 300 mM NaCl, 10% glycerol, and 30 mM imidazole), bound proteins were eluted with elution buffer (50 mM Tris, pH 8.5, 10 mM MgCl_2_, 250 mM NaCl, 10% glycerol, 250 mM imidazole) and collected (1 mL fractions). Fraction protein content was measured via a Bradford assay. The most concentrated fractions were pooled and dialyzed overnight into 4 L of dialysis buffer (50 mM Tris, pH 8.5, 10 mM MgCl_2_, 250 mM NaCl, 20% glycerol, 1 mM TCEP). Protein purity was assessed via SDS/PAGE and Coomassie staining.

### 4.3. PHGDH Assay

The enzymatic assay was adapted from a previously described procedure [25]. In brief, the reactions were performed in PHGDH assay buffer (50 mM Tris, pH 8.5, and 1 mM EDTA) following resorufin fluorescence emission (Ex 550 nm/Em 580 nm) over time. Substrate and enzyme concentrations were as follows: 240 μM 3-PG; 120 μM NAD^+^; 30 mM glutamate; 0.1 mM resazurin; 12 ng/μL human PHGDH; 20 ng/μL human PSAT1; 0.18 µg/µL diaphorase. Inhibitors concentration varied from 0.03 µM to 1000 µM. The final concentration of DMSO in the assay mixture was set to 5%.

### 4.4. Target Engagement in Cell Lysates

Target engagement in cell lysates was determined by the cellular thermal shift assay (CETSA) using cell lysates. Therefore, HL-60 cells were harvested by centrifugation, washed in PBS, and resuspended in TBS-T to a density of 1.67 × 106 cells/mL. Cells were lysed with three freeze-thaw cycles (3 min/3 min at −80 °C/37 °C). The lysate was clarified by centrifugation at 17,000× *g* for 20 min at 4 °C. The supernatant was aliquoted into PCR tubes (60 mL/tube), and 0.5 mL DMSO (0.8% *v*/*v* final) or 83 mM compound was added to the supernatant and mixture incubated for 30 min at room temperature. The lysates were then heated at 60 °C (or 37 °C for DMSO reference sample) for 3 min in an Applied Biosystems Veriti Thermal Cycler, followed by cooling for 3 min at 4 °C. In order to pellet protein aggregates, the samples were centrifuged at 20,000× *g* for 20 min at 4 °C. Forty five milliliters of clarified supernatant was mixed with 15 mL 4 × Laemmli buffer and heated for 10 min at 95 °C. Fifteen milliliters of the mixture was separated by SDS-PAGE on a Mini-PROTEAN TGX or Criterion TGX gel (4%–15%) and transferred onto nitrocellulose membrane using a Trans-Blot Turbo Transfer System (BioRad, Hercules, USA). Membranes were blocked in 1:1 Odyssey blocking buffer (PBS)/PBS for 1 h at room temperature and incubated with primary antibody at 4 °C overnight. Primary antibodies for PHGDH (HPA021241, Sigma Aldrich (Overijse, Belgium), rabbit antibody), PSAT (ab96136, abcam, rabbit antibody) and Vinculin (H-10) (sc-25336, Santa Cruz, goat antibody), were diluted 1:1000 (PSAT and PHGDH) or 1:2000 (Vinculin) in 1:1 Odyssey blocking buffer (PBS)/PBS. Secondary antibody incubation was performed for 1 h at room temperature with anti-rabbit or anti-goat antibody (IRDye 800 CW) at 1:10,000 dilution in 1:1 Odyssey blocking buffer (PBS)/PBS. Protein bands were visualized with an Odyssey Fc Imager and analyzed with Image Studio Software (LiCor Biosciences, Lincoln, USA).

### 4.5. Cell Models and Cytotoxicity Assay

BT-20, MDA-MB-468 breast cancer cells, and BJ-5ta were purchased from ATCC and cultured in DMEM medium supplemented with 10% heat-inactivated fetal bovine serum (FBS) and 1% penicillin/streptomycin. For the cytotoxicity assay, cells were seeded at low density in 96-well plates in serine containing media following a previously reported procedure [25]. Media was then aspirated, and cells were incubated in fresh serine-replete or -deplete media containing compounds at indicated concentrations or vehicle (DMSO). Cells were grown for 5 days at 37 °C with drug and media changed daily before assaying cell viability using a Presto Blue assay (Life Technologies) according to the manufacturer’s instructions.

## Figures and Tables

**Table 1:** Michaelis–Menten enzyme kinetics for PHGDH and PSAT1. All experiments were performed with at least triplicates and K_m_ values were determined in two or more independent experiments.

	PHGDH		PSAT1	
	3-PG	NAD^+^	3-PSer	α-KG
Experimental K_m_	294.3 ± 12.4 μM	30.4 ± 4.3 μM	25.1 ± 5.3 μM	35 ± 4.2 μM
K_m_ from literature	260 µM [26]	22 µM [26]	5 µM [27]	800 µM [28]

**Table 2:**
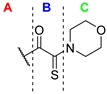
Results of PHGDH inhibition for compounds **1**–**11**. All experiments were performed with at least triplicates and IC_50_ values were determined in two or more independent experiments. * Compounds previously published in Ravez et al. [25].

	Part A	PHGDH IC_50_ (µM)
**1**	Phenyl *	111.1 [87.3–131.2]
**2**	4-Chlorophenyl *	20.3 [15.6–26.4]
**3**	3-Chlorophenyl *	>150
**4**	2,4-Dichlorophenyl	77 [65.3–98.6]
**5**	Cyclohexyl	>150
**6**	4-Pyridine	>150
**7**	3,4-Dichlorophenyl	>150
**8**	2-Naphtyl	>150
**9**	Ethyl	>150
**10**	Adamantyl	>150
**11**	*t*-Butyl	>150

**Table 3:**
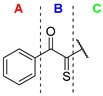
Results of PHGDH inhibition for compounds **12**–**21**. All experiments were performed with at least triplicates and IC_50_ values were determined in two or more independent experiments.

	Part C	PHGDH IC_50_ (µM)
**12**	Pyrrolidine	>150
**13**	4,4-Difluoropiperidine	>150
**14**	4-Methylpiperidine	85.1 [37.8–101.2]
**15**	Piperidine	>150
**16**	Diethylamine	>150
**17**	Thiomorpholine	>150
**18**	*N*-methylpiperazine	>150
**19**	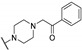	>150
**20**	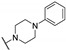	>150
**21**	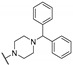	>150

**Table 4:**
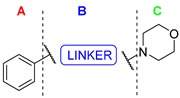
Results of PHGDH inhibition for compounds **22**–**37**. All experiments were performed with at least triplicates and IC_50_ values were determined in two or more independent experiments.

	Part B	PHGDH IC_50_ (µM)
**22**	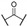	>150
**23**	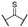	106 [63.2–178.8]
**24**	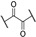	>150
**25**	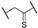	>150
**26**	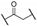	>150
**27**	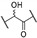	>150
**28**	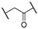	>150
**29**	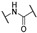	>150
**30**	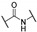	>150
**31**	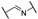	>150
**32**	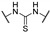	>150
**33**	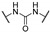	>150
**34**	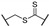	>150
**35**	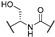	>150
**36**	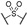	>150
**37**	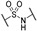	92.1 [58.4–145.2]

**Table 5:** Cell proliferation inhibition data (IC_50_). All experiments to determine IC_50_ values were performed with at least triplicates at each compound dilution, and all IC_50_ values were averaged when determined in two or more independent experiments. * Values previously published in Ravez et al. [25].

	Tumor Cell Lines		
	PHGDH-Dependent	PHGDH-Independent	Normal Cell Line
	BT-20	MDA-MB-231	BJ-5ta
1	67.8 µM	>100 µM *	>100 µM
2	21.3 µM	>100 µM *	>100 µM
38	42.2 µM	>100 µM	>100 µM
39	>100 µM	>100 µM	>100 µM
NCT-503	6.52 µM	>100 µM	>100 µM
